# Variation in Lipid Peroxidation in the Ejaculates of Wild Banded Mongooses (*Mungos mungo*): A Test of the Oxidative Shielding Hypothesis

**DOI:** 10.3390/antiox13091124

**Published:** 2024-09-18

**Authors:** Graham Birch, Magali Meniri, Chris Mitchell, Francis Mwanguhya, Robert Businge, Solomon Ahabyona, Hazel J. Nichols, Michael A. Cant, Jonathan D. Blount

**Affiliations:** 1 Centre for Ecology & Conservation, Faculty of Environment, Science & Economy, University of Exeter, Penryn Campus, Cornwall TR10 9FE, UK; 2 Banded Mongoose Research Project, Mweya Village, Queen Elizabeth National Park, Kasese District, Uganda; 3 Department of Biosciences, Swansea University, Singleton Campus, Swansea SA2 8PP, UK

**Keywords:** cooperative breeder, intergenerational effects, life-history, oxidative stress, reproductive costs

## Abstract

Reproductive activity is costly in terms of future reproduction and survival. Oxidative stress has been identified as a likely mechanism underlying this cost of reproduction. However, empirical studies have yielded the paradoxical observation that breeders often sustain lower levels of oxidative damage than non-breeders. The oxidative shielding hypothesis attempts to explain such data, and posits that breeders pre-emptively reduce levels of oxidative damage in order to protect their germ cells, and any resultant offspring, from harm caused by exposure to oxidative damage. While there is some empirical evidence of oxidative shielding in females, there have been no explicit tests of this hypothesis in males, despite evidence of the oxidative costs to the male reproductive effort and the vulnerability of sperm cells to oxidative damage. In this study, we assess lipid oxidative damage (malondialdehyde, MDA) in the ejaculates of reproducing and non-reproducing wild banded mongooses. We found that, among breeding males, ejaculate MDA levels were lower during mate competition compared to 2 months later, when individuals were not mating, which is consistent with the oxidative shielding hypothesis, and similar to findings in females. However, ejaculate MDA levels did not differ significantly between breeding and non-breeding individuals at the time of mating, contrary to expectation. The finding that ejaculate MDA was not higher in non-breeders may reflect individual differences in quality and hence oxidative stress. In particular, breeders were significantly older than non-breeders, which may obscure differences in oxidative damage due to reproductive investment. Further research is needed to establish the causal relationship between reproductive investment and oxidative damage in ejaculates, and the consequences for offspring development in banded mongooses and other species.

## 1. Introduction

Finite resources force resource allocation trade-offs [1]. Underpinning life-history theory is the trade-off between an individual’s reproductive investment and their future fecundity and survival [2,3,4,5]. Oxidative stress has been proposed as a proximate mechanism that underlies life-history trade-offs [2,6,7,8]. Metabolic activity, for example, is associated with reproduction, growth or mate competition, and generates reactive oxidative species (ROS) which the body can attempt to neutralise using antioxidant defences. If these defences are breached, however, a state of oxidative stress arises where critical biomolecules such as lipids, protein, and DNA incur oxidative damage. Oxidative stress is associated with survival decline and accelerated senescence of tissues [2,3,9,10]. There is plenty of evidence that oxidative damage increases with reproductive effort [3,4,11,12]. However, a meta-analysis has identified that oxidative damage in breeders is often below the damage seen in non-breeders [13]. This paradoxical reduction in oxidative damage associated with the reproductive state may only make sense when considering life-history theory through an intergenerational lens. Oxidative stress can have consequences that transmit across generations; ROS and oxidised biomolecules can pass to offspring through germline tissue [13,14], the placenta [15,16], and in milk [17,18], causing intergenerational oxidative damage (IOD). Exposure of offspring to oxidative damage during early development could have serious consequences for fitness [19]. Diminishment of oxidative damage by breeders has been suggested to act as an adaptive, pre-emptive measure to protect the next generation from oxidative insults (the oxidative shielding hypothesis) [13].

Explicit tests of the oxidative shielding hypothesis have so far only been reported in females [12,20,21]. This sex bias in studies may largely be due to the expectation that females invest more in reproduction than males [4], and are therefore under greater selection pressure to mitigate a higher potential for oxidative harm to offspring. However, there are, in fact, considerable oxidative costs of mate competition faced by males (reviewed in [22]). The oxidative costs of mate competition arise through physical activity [23]. For example, physical contests over harems in male elephant seals (*Mirounga angustirostris*) are associated with plasma oxidative damage to lipids and DNA [24], although evidence of IOD in the germline was not assessed in this study. Other oxidatively costly forms of male competition have been associated with germline damage, including sexual signalling, where diversion of carotenoids from oxidative defence to bolster colouration was associated with sperm cell DNA damage in sticklebacks (*Gasterosteus aculeatus*) [25]. Similarly, higher metabolic activity necessary to power competitive sperm cell phenotypes [26] was found to be associated with DNA damage in the sperm cells of zebrafish (*Danio rerio*) [27]. 

Sperm cells are defended against oxidative damage by the presence of antioxidant enzymes such as superoxide dismutase (SOD) and glutathione peroxidase (GPx) in seminal fluid [26,28]. The dietary antioxidant vitamin E is present within sperm cells and is particularly important in preventing lipid oxidative damage in cell membranes [29,30,31]. However, sperm may be particularly vulnerable to the oxidative costs of reproduction; unlike somatic cells and oocytes, they lack intracellular antioxidant enzymes, such as SOD, GPx, and catalase (CAT) [32,33,34,35,36], while also having limited DNA repair pathways [32,37,38]. If antioxidant defences become overwhelmed by ROS and the male germline incurs oxidative damage, offspring can suffer from a wide range of pathologies, such as developmental disorders, cancer, and birth defects [32,39,40,41,42,43,44,45,46,47], ultimately reducing survival [48,49]. These pathologies are despite the millions of sperm cells produced in each ejaculate, indicating that the fertilization race often does not filter out oxidatively damaged cells. The potentially damaging consequences of paternally derived IOD for offspring suggest that oxidative shielding should be selected for in breeding males. We are only aware of one previous study that has assessed IOD due to reproduction in males. Although the authors seemed unaware of the oxidative shield hypothesis, they did find evidence suggesting IOD is mitigated: field crickets (*Gryllus bimaculatus*) undergoing mate competition were found to have reduced oxidative damage in their sperm compared to males that experienced no mate competition [50]. 

We aimed to test the oxidative shielding hypothesis in wild male banded mongooses (*Mungos mungo)*. This species is a medium reproductive skew cooperative breeder, with multiple reproducing males and females present in each group. Groups reproduce year-round, with short discrete bouts of mate competition occurring when all reproducing females in a group synchronously enter oestrus over a few days [51]. Reproducing males guard individual females during these group oestrus events, continuously shadowing their focal female over the few days they remain sexually receptive, and aggressively preventing copulations with rival males [52]. We can reason this short window means sperm cells stored in the epididymis, rather than spermatogonial stem cells still being formed, would be most culpable for any IOD associated with reproductive effort. As we focus on males, these group oestrus events are referred to as ‘mate competition events’. Guarding is associated with activity costs such as weight loss [53]. We can assume that guarding, as a component of reproductive expenditure, also carries an oxidative cost [23]. Although additional males may occasionally succeed in sneaking copulations (‘pesterers’; [52]), the rest of the mature males in the group do not engage in any reproductive activity. These non-reproductive males provide a natural control group that should not incur reproductive activity costs and should not express an oxidative shielding response. We first compared lipid oxidative damage levels in the ejaculates of breeding and non-breeding males during the mate competition period. We then assessed how this lipid oxidative damage changed within breeding and non-breeding individuals over time, comparing the period of mate competition with a period 2 months after mate competition. Oxidative shielding would be indicated by lower levels of lipid oxidative damage in breeders compared to non-breeders during the mate competition period. Oxidative shielding would also be indicated by a breeder-specific increase in oxidative damage from this mate competition for the period 2 months after competition, as oxidative defence mechanisms should become relaxed once the prospect of passing damage to the next generation has passed. Lastly, we attempted to assess the relationship between ejaculate oxidative damage levels and the survival of offspring sired by this sample of males. 

## 2. Methods

### 2.1. Study Population

Data were collected on a wild banded mongoose population living on the Mweya Peninsula, Queen Elizabeth National Park, Uganda. Comprehensive life-history data have been collected for this population since 1995, including births and deaths. The age of all individuals sampled in this study was known. A genetic pedigree based on 43 microsatellite loci has been collected from 2003 to 2020 (see references for how the pedigree is obtained [54,55,56]). Groups are highly male-skewed due to female-biased mortality and eviction events commonly targeted at females [51]. Compared to females that typically start reproducing at one year of age, reproductive skew delays the onset of breeding in males due to waiting in a ‘queue’ for reproductive positions [52] leading to group demographics made up of a number of reproducing and non-reproducing adult males. 

### 2.2. Experimental Design for Testing the Oxidative Shielding Hypothesis

Ejaculate samples were taken from banded mongoose males between May 2020 and January 2023. Up to 2 reproducing males (guarders) and 2 non-reproductive males were sampled in each group repeatedly (n reproducing males = 13 (39 total samples), n non-reproducing = 14 (26 total samples)). An optimal experimental design to test the oxidative shielding hypothesis required collecting samples on the same individuals before, during and after reproduction. However, in banded mongoose groups, there is no pause between pup care and the next mate competition period. All sampling periods outside of mate competition were close to past and future mate competition events, therefore a functional difference between the periods before and after reproduction was less applicable. Instead, only two sampling periods were used: (1) during group mate competition; and (2) 2 months after mate competition (just before the birth of the resulting offspring). 

### 2.3. Ejaculate Collection

Males were caught using baited Tomahawk traps (Tomahawk Live Trap, Hazelhurst, WI, USA). Isoflurane (5%) (IsoFlo, Abbott Laboratories, Chicago, IL, USA) was used to anesthetise males, reduced to 2% once they lost consciousness. Ejaculates were collected using electro-ejaculation [57]. The male was placed on its back and the penis was cleaned gently with wetted cotton wool. Aqueous lubricant (K-Y; Johnson and Johnson, New Brunswick, NJ, USA) was inserted into the rectum with a pipette. A probe covered in the same lubricant was inserted 1.5 cm into the rectum. Two electrodes at the end of the probe faced upwards to stimulate the prostate gland. A series of 5 electrical stimulations were transmitted through the probe using an audio amplifier (QTX KAD-2BT; AVSL Group Ltd., Manchester, UK). Each series comprised 17 half-second bursts with half-second breaks in between, that progressively increased in intensity (0.5–5 mA; see Supplementary for full song details), controlled with Audacity software version 2.21 (Microsoft). A multimeter (Kewtech KT117, Sunningdale, UK) was monitored throughout to ensure the current remained within the expected range. Stimulation stopped for 15 s between each series. All ejaculates produced over the 5 series of stimulations were collected. Ejaculates were handled with wooden cocktail sticks. A small amount of sample, approximately 2 μL, was smeared onto a slide to approximate sperm cell counts under an Olympus CX43 phase contrast microscope (Gtvision, Newmarket, UK). Cell counts for 23/63 samples could not be estimated due to inadequate sample volumes. To prevent artefactual oxidative damage, the remainder of the ejaculates (not used for cell counts) was immediately transferred into screw-top tubes and flash frozen in liquid nitrogen to be sent back to the UK in an insulated cryogenic shipper (Taylor-Wharton CX500; Wolflabs, Pocklington, York, UK) for subsequent MDA analysis. After sampling, males were returned to their group and released. 

### 2.4. Sample Preparation and MDA Analysis

To homogenise, ejaculate samples were suspended in double quantities of phosphate-buffered saline (PBS) (1 mg sample:2 μL PBS). Zirconia beads were added to break apart the suspended sample using an Omni bead ruptor running for 1 min. Ejaculate malondialdehyde was determined using high performance liquid chromatography (HPLC) with fluorescence detection [58] with some modifications, as we have described previously [12]. HPLC grade chemical solutions were prepared using Milli-Q water. An aliquot (10 µL) of ejaculate solution or standard (1,1,3,3-tetraethoxypropane, TEP), 10 µL 0.05% butylated hydroxytoluene solution in 95% ethanol, and 80 µL of 0.44 M phosphoric acid solution were added to a 2 mL screw-cap reaction tube. An amount of 20 µL of 42 mM 2-thiobarbituric acid (TBA) was added to initiate the reaction. Tubes were capped and vortexed, before incubation on a dry heat block for 1 h at 100 °C to allow formation of MDA-TBA adducts. The reaction was stopped by placing the samples on ice for 5 min. Butanol (80 µL) was added, and tubes were vortexed for 20 s. Samples were then centrifuged at 13,000× *g* for 3 min at 4 °C. A 50 µL aliquot of the upper butanol phase was transferred to a 0.3 mL crimp top HPLC vial. Samples (20 µL) were injected into an Agilent HPLC system (Agilent technologies Inc., Santa Clara, CA, USA) fitted with a Hewlett-Packard Hypersil 5µ ODS 100 × 4.6 mm column. The mobile phase was a methanol-buffer, the buffer being a 50 mM anhydrous solution of potassium monobasic phosphate at pH 6.8 (adjusted using 5 M potassium hydroxide solution), running isocratically over 3.5 min at a flow rate of 1 mL min^−1^. The column oven was set at 37 °C. Peaks were collected using a fluorescence detector (RF2000; Dionex, Sunnyvale, CA, USA), with excitation and emission wavelengths of 515 nm and 553 nm, respectively. Peaks were quantified relative to an external calibration curve prepared using a TEP stock solution (5 µM in 40% ethanol) serially diluted using 40% ethanol to give known values in the range 0–5 µM.

### 2.5. Statistical Analysis of Oxidative Shielding

We planned to focus on cross-sectional comparisons of MDA concentrations, such as comparing breeders and non-breeders in the same mate competition event, and within-individual changes between the period of mate competition and the period following 2 months after the mate competition period. However, we had problems with the collection of analysable ejaculate samples. Samples were often too small in volume or not produced at all. In the 15 mate competition periods that yielded successful MDA measures, only 7 had MDA comparisons between at least one breeding and non-breeding individual within the same competition event. Similarly, for the 23 events that had successful MDA measures either in the mate competition period or the period 2 months post the same mate competition, there were only 2 individuals, both breeders, that had MDA comparisons between these periods within the same event. Indeed, there were only 9 individuals (7 breeders and 2 non-breeders) that had mating and non-mating MDA comparisons across mate competition events. Because of these issues, we were forced to make non-paired comparisons of MDA measures across different males rather than within individuals, and across different mate competition events rather than within the same event. 

Three general linear models (GLMs) were run using Bayesian inference (JAGS MCMC; chains = 3, iterations = 10,000, burnin = 1000, thinning internal = 100). All models used uninformed priors. Convergence of chains for each parameter was confirmed using Rhat values (<1.1 for successful convergence) and traceplots using the R2JAGS package. 

The initial two GLMs modelled MDA concentration with a Gaussian error distribution. A square root transformation was used to normalise moderately right skewed MDA concentrations. The first of these two models regressed the molar concentration of MDA (μm) in ejaculates collected during the mating period (n samples = 31) against the reproductive state of the male (breeder versus non-breeder). Out of 31 ejaculate samples, 12 did not have associated slide smears with sperm cell counts, 14 had smears with no sperm cells, and 5 smears had cells. To account for an effect of the presence of sperm cells on MDA levels, cell presence was fitted as a 3-level categorical variable (cells not counted, cells absent, cells present). The 3-level categorical term was fitted with ‘cells absent’ as the intercept for comparison. To control for repeated sampling on the same males and non-independent environments for males living in the same group and sharing the same mate competition event, random intercepts for male ID, group ID, and event ID were fitted. This included 18 unique males from 4 unique groups participating in 15 unique mate competition events. 

The second model regressed the molar concentration of MDA (μm) in ejaculates collected during the mating period and 2 months after the mating period (n = 63 samples) against reproductive state of each male and the sampling period (mating period versus non-mating period) including an interaction term. Similar to the first model, cell presence as a 3-level categorical variable (n cells not counted = 23, n cells absent = 30, cell present = 10) and random intercepts for male ID (n = 24), group ID (n = 4), and event ID (n = 23) were fitted into the second model. MDA concentration comparisons between sampling periods, where the non-mating period was 2 months after the mating period, were given the same event ID. 

In a third model using a Bernoulli error distribution, we regressed the probability each sampled male was a breeder, as opposed to a non-breeder during an oestrus event (n = 27 cases), against their age in years. Random intercepts for male ID (n = 14), group ID (n = 4), and event ID (n = 14) were fitted.

A fourth model was planned to assess the evidence of an intergenerational effect of MDA in fathers’ ejaculates on the survival (to 6 months of age) of pups that they sired. However, sampling problems prevented a formal analysis (see Supplementary Data). 

### 2.6. Ethics

Data were collected under license from the Uganda National Council for Science and Technology, and all research procedures were approved by the Uganda Wildlife Authority. All procedures adhered to the ASAB Guidelines for the Treatment of Animals in Behavioural Research and Teaching and were approved by the Ethical Review Committee and Animal Welfare Review Board of the University of Exeter (eCORN000006).

## 3. Results

**Figure 1:**
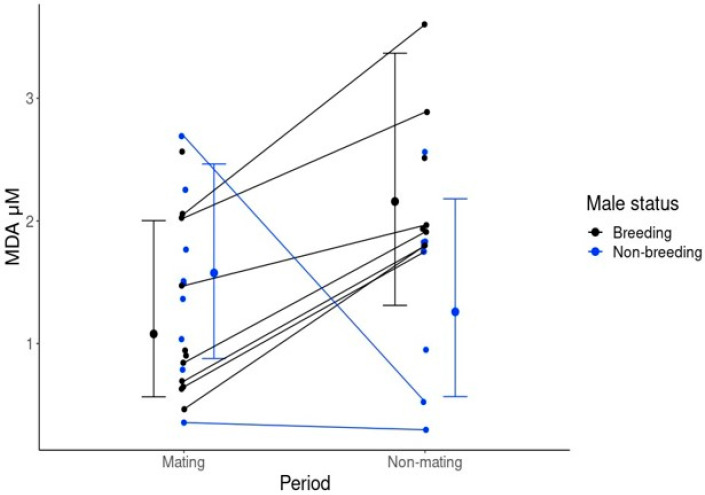
The effect of breeding status of a male and the time period on MDA concentration in ejaculates. Error bars represent the mean, lci and hci of the predicted posterior distribution of model b (Table 1). Points show 32 mean MDA concentrations for each male across mate competition periods and of breeding and non-breeding reproductive status. Lines indicate individuals where comparisons were possible between mating and non-mating periods across all mate competition events (7 for breeders, 2 for non-breeders).

**Figure 2:**
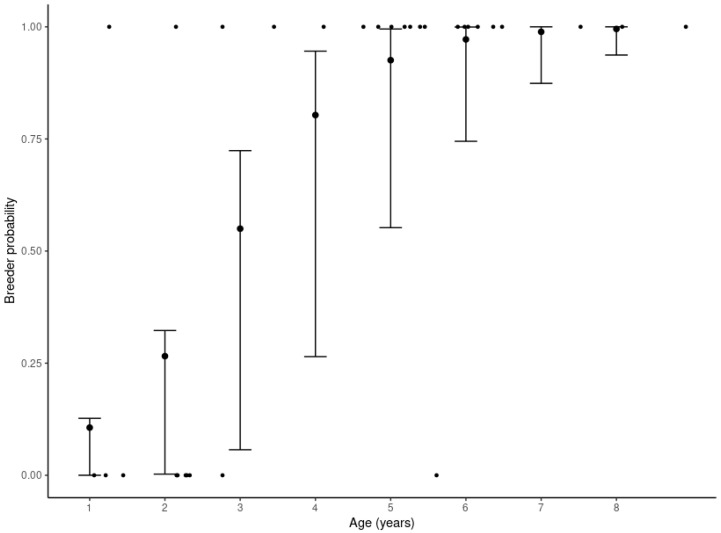
The effect of age of a sampled male on their probability of breeding during a given oestrus period. Error bars represent the mean, lci and hci of the predicted posterior distribution of model c (Table 1). Large points mean probabilities predicted by the model. Small points represent raw data for when individuals of a given age were a breeder (1) or a non-breeder (0).

There was no significant difference in ejaculate MDA concentrations between breeders and non-breeders among mate competition periods (Table 1a). However, there was a significant interaction between the reproductive state of the male and the sampling period on MDA levels in ejaculates (Table 1b: MS:GP). The interaction indicates an effect of the period of sample collection on MDA levels across breeding males, but not across non-breeding males (Figure 1). MDA increased in breeders from a mean of 1.059 μM (lci = 0.443 μM, hci = 1.813 μM) during mate competition events to 2.194 μM (lci = 1.079 μM, hci = 3.275 μM) during the pregnancy period (Figure 2), compared to no change in MDA in non-breeders (mate competition period: mean = 1.498 μM, (lci = 0.537, hci = 2.460 μM); pregnancy period: mean = 1.267 μM, (lci = 0.525, hci = 2.142)). The presence of cells or unknown cell counts had no effect on MDA in ejaculates compared to ejaculate samples where cells were absent in either model (Table 1b). 

There was a significant positive effect of age on the probability a sampled male was a breeder during an oestrus period (Table 1c, mean = 1.4, lci = 0.61, hci = 2.36). For example, a 1-year old male has a lower breeding probability (Figure 2; mean = 0.11, lci = 0.01, hci = 0.36), compared to a 4-year old male (mean = 0.80, lci = 0.51, hci = 0.97).

## 4. Discussion

We found some evidence in support of the oxidative shielding hypothesis in male banded mongooses. Compared to post-mate competition, levels of oxidative lipid damage in ejaculates during mate competition were lower in reproducing, but not non-reproducing, male banded mongooses. This is consistent with oxidative shielding, suggesting that ROS production in sperm is decreased and/or antioxidant defences in ejaculates may be up-regulated during mate competition to protect the next generation from IOD [22]. By contrast, there was no significant difference in oxidative damage between reproducing and non-reproducing males during mate competition. This result is inconsistent with the oxidative shielding hypothesis, which predicts that reproducing males should have lower levels of oxidative damage than non-reproducing males during mating [13,22]. However, the lack of a significant effect of reproductive state in this analysis of banded mongooses seems likely to reflect differences in quality between breeders and non-breeders, as suggested by our finding that breeding individuals were older than non-breeders. 

Significant oxidative costs of reproduction without shielding could cause offspring to suffer from IOD during critical early development with later consequences for their reproductive fitness [48,49]. Reproductive activity may be costly in two ways in male banded mongooses. First, there are likely oxidative costs of physical activity associated with mate competition, which incur increased ROS production [23]. Second, there may be opportunity costs of diverting time away from necessary activities such as foraging. Such activity costs may cause large decreases in body condition, as reported in territorial patrolling anole lizards (*Anolis gundlachi*) [60]. Vitamin E, one of the few antioxidants present in sperm cells, is diet-derived and may itself be in limited supply [29,30,31]. Therefore, guarding may cause an increased potential for oxidative damage as a result of increased ROS production, while there may be associated depletion of antioxidant defences. To protect the next generation, those antioxidant defences that are available while breeding may be best allocated to protect the germline through an oxidative shielding response. The exhaustion of antioxidants after oxidative shielding may explain the increase in oxidative damage in breeders, compared with non-breeders, after mate competition. If oxidative shielding underlies these results, then banded mongooses would be the first species for which there is evidence to suggest potential oxidative shielding responses in both sexes. Oxidative shielding in males would be despite oocytes having the ability to repair some paternal DNA damage [49], and oocytes having more varied antioxidant defences [33,61]. 

We can suggest two potential explanations for why ejaculate oxidative damage levels did not differ significantly between breeding and non-breeding males during mate competition. Firstly, it is possible that male banded mongooses do not exhibit an oxidative shielding response when breeding. An absence of a shielding response could be explained by a lack of significant oxidative costs of mate competition compared to regular levels of physical activity when not reproducing, in which case an increased antioxidant defence of ejaculates may not benefit the next generation [22]. Any oxidative damage to DNA that is passed through the germline could potentially be repaired by oocytes [49], which may reduce the need for males themselves to mitigate IOD. However, we think this explanation is unlikely given our finding that ejaculate MDA levels in breeders were lower during mating than two months after mating. Secondly, a more plausible explanation is that there are inherent quality differences between breeding and non-breeding males, such that breeders may have relatively high levels of oxidative damage even when mating. 

Reproducing males are high quality individuals, because to hold reproductive positions in these groups successfully requires having the condition to defend access to reproducing females from rival males [52]. Indeed, we found that breeders were significantly older than non-breeders. Age is strongly associated with body size and competitive ability in male banded mongooses [52]. These higher quality individuals are likely to have an advantage facing condition-dependent life-history trade-offs [62], and may be more resistant than lower-quality males to survival or reproductive costs that would result from high levels of oxidative damage. Resistance to oxidative costs may allow these higher-quality individuals to participate more often in oxidatively costly activities. For example, in intergroup interactions, older males show more aggression during an experimental presentation of outgroup members [63], and they are the most influential group members in determining the outcomes of wars with rival groups [64]. Older males are also more active in allomarking behaviour [65]. Through greater resistance to the consequences of oxidative damage, we may expect these higher-quality males on average to have higher levels of oxidative damage throughout the year compared to non-breeders. A shielding response in such males may reduce oxidative damage to levels similar to those seen in non-breeders. To test this idea definitively would require the longitudinal sampling of males, which was not possible in our study. Specifically, we had much less success sampling non-breeding individuals repeatedly across the two ejaculate collection periods, and variation among individual non-breeders was largely not controlled when making MDA comparisons during and outside of mate competition. 

Additionally, breeding males should have higher circulating levels of androgens than non-breeders, which might in part mask any oxidative shielding response. This is because elevated androgen levels have been linked to increased ROS production due to anabolic effects [66,67]. However, there is also evidence that elevated androgens are associated with enhanced antioxidant protection of sperm [68,69,70]. Further research will be needed to evaluate the relationships between breeding status, androgen levels, and oxidative stress.

Previous research in banded mongooses has shown elevated blood plasma levels of vitamin E in males during the pregnancy period (on average a month after mate competition) compared to samples taken 3 months before or after [12]. Although no samples were collected during mate competition, this elevation of vitamin E close to mate competition, relative to other comparison times, points to the possibility of elevated somatic antioxidant protection during male reproductive activity. If circulating vitamin E levels are mirrored in the ejaculate during mate competition it would suggest vitamin E is involved in oxidative shielding responses. We were not able to measure vitamin E levels in ejaculates in the present study due to insufficient sample volumes. However, a recent study of the Mediterranean field cricket showed a reduction in protein oxidative damage and increased antioxidant capacity in ejaculates during sperm competition [50]. It would also have been interesting to assess DNA oxidative damage in sperm cells as this has direct implications for offspring fitness [71]. Unfortunately, we were unable to extract DNA from ejaculate samples successfully, likely due to the absence of sperm cells in 75% of samples and the small sample volumes. The median ejaculate mass was 33 mg, and out of 96 ejaculates, 33 samples weighed less than 10 mg and could not be used. These samples also had sticky, globular properties making them difficult to homogenise in a solution [72]. These problems with our samples meant that we were restricted to measuring lipid oxidative damage in the form of MDA and could not also measure the antioxidant levels and any oxidative DNA damage. 

Reductions in oxidative stress in breeders are predicted to benefit their offspring [13,22]. Therefore, an important challenge for further research is to attempt to detect any intergenerational effects of fathers’ ejaculate oxidative damage levels on the development, survival, and future fecundity of their offspring. This is a difficult task in study systems in the wild. It requires an intact genetic pedigree to confirm the paternity of offspring, and continued observations of the next generation to observe their development, survival, and reproductive success. Such work requires long-term funding and an intensive field sampling effort. In our case, sampling the male required removing them from the group during the mate competition period. Although these males were only taken from the group for a few hours, during this time they may have lost out to competitors, which may have contributed to the low number of pups that could be confirmed as sired by these males in our genetic pedigree; this may also have contributed to our inability to conduct a formal analysis of any intergenerational effects (see Supplementary Data). Depending on the average lifespan and natural mortality in the study system, there is also a long lead time to obtaining fitness data for the next generation with enough variation to analyse. For example, from our dataset, 2/11 pups are now dead; we will not know the lifetime reproductive success of the other 9 pups for many more years. Where these challenges can be overcome, evidence of an intergenerational effect of fathers’ ejaculate oxidative damage levels on offspring fitness is necessary to validate the oxidative shielding hypothesis.

The complex properties of banded mongoose ejaculates may have consequences for oxidative shielding responses. Mammalian ejaculates can be made up of discrete fractions formed within different tissues such as accessory glands compared to the testis [73]. These fractions vary in their composition and cell densities [74,75,76]. The ejaculates of banded mongooses may have additional complexity as their properties are consistent with copulatory plugs [72,77]. Copulatory plugs are structurally complex, formed by a network of crosslinked-molecules that underpin their viscous and coagulated properties [72]. The function of an ejaculate with copulatory plug properties may be split between different fractions [78]. For example, in guinea pigs (*Cavia porcellus*), plugs did not induce pregnancy themselves when collected and transferred to a second female, suggesting viable cells may be released from separate fractions of the ejaculate [79]. Anecdotally from our study, slide smears showed inconsistencies in the presence of sperm cells in ejaculates collected from the same electro-ejaculation run (same male), suggesting the concentration of sperm cells varies throughout the banded mongoose ejaculate. This inconsistency may account for the 75% of samples with no apparent sperm cells; for example, if only a copulatory plug rather than functionally fertile fraction of the ejaculate was collected during electroejaculation. The regulation of oxidative defences can differ between tissues. For example, there is evidence of prioritisation of oxidative defence in sperm versus blood plasma in house sparrows (*Passer domesticus*) [80]; the opposite has been shown with somatic erythrocytes protected over ejaculates in Damaraland mole-rats (*Fukomys damarensis*) [81]. There is evidence of regulatory differences in oxidative defences within ejaculates between fractions [82,83], and conceivably oxidative shielding mechanisms may be prioritised in the fractions of the ejaculate with more viable sperm cells compared to more disposable fractions important in forming a copulatory barrier itself. This deserves further study. 

## 5. Conclusions

We found some evidence of oxidative shielding in banded mongooses, specifically a reduction in lipid oxidative damage (MDA) in reproducing males during mate competition events. Future work is necessary to build a more robust picture of oxidative shielding responses in males; this should include studies of the association between ejaculate levels of oxidative damage and offspring development and survival. Our results add to the considerable body of comparative evidence of lower levels of oxidative damage in breeding compared with non-breeding females [12,20] The extent to which oxidative shielding may be a joint endeavour carried out by both sexes remains to be seen.

## Figures and Tables

**Table 1:** Model outputs for predicting MDA oxidative damage in male ejaculates.

Model	Term	Effect	sd	2.50%	50%	97.50%	Rhat	f	Overlap0
a	Intercept	1.30	0.24	0.85	1.29	1.79	1.02	1.00	Yes
	Male status (MS)	−0.19	0.15	−0.48	−0.19	0.09	1.00	0.89	Yes
	Cells present	−0.05	0.20	−0.41	−0.04	0.37	1.01	0.57	Yes
	Cells not counted	−0.16	0.20	−0.56	−0.15	0.22	1.00	0.80	Yes
	Random effect	Group	Male.ID	Event					
	sd	0.038	0.079	0.084					
b	Intercept	1.23	0.23	0.78	1.23	1.73	1.02	1.00	+
	Male status (MS)	−0.20	0.14	−0.47	−0.20	0.05	1.00	0.92	Yes
	Collection period (CP)	−0.11	0.15	−0.39	−0.11	0.16	1.00	0.78	Yes
	**MS:CP**	**0.55**	**0.18**	**0.23**	**0.55**	**0.89**	**1.00**	**1.00**	**+**
	Cells present	−0.01	0.14	−0.31	−0.01	0.26	1.01	0.54	Yes
	Cells not counted	0.01	0.10	−0.20	0.01	0.18	1.01	0.56	Yes
	Random effect	Group	Male.ID	Event					
	sd	0.02	0.078	0.015					
c	Intercept	−4.05	1.59	−7.13	−4	−1.27	1.01	1	-
	**Age**	**1.4**	**0.47**	**0.61**	**1.4**	**2.36**	**1.01**	**1**	**+**
	Random effect	Group	Male.ID	Event					
	sd	0.08	0.11	0.11					

Model output for ejaculate MDA comparisons between: (a) breeders and non-breeders during mate competition; (b) ejaculate MDA comparisons between breeders and non-breeders across mate competition and 2 months post-competition periods; and (c) the effect of age on the probability a sampled male is a breeder. Means (effect), credible intervals (0.025, 0.975), and median (50%) effects for each covariate are sampled from the untransformed posterior distribution of each model. Effect sizes are on the square root scale. f is the proportion of the posterior distribution with the same sign as the mean. Overlap 0 shows whether 0 overlaps with the range of 2.5% and 97.5% quantiles of the posterior distribution for each fitted parameter, with bold covariates those with significant effect (no overlap). Where there was no overlap, the direction of the effect is given in ovelap0. Rhat is a measure of chain convergence (<1.1) [59]. The standard deviation of the three random effects, Group, Male ID, and event ID is given for each model.

## Data Availability

The research data supporting this publication are openly available from the University of Exeter’s institutional repository at https://ore.exeter.ac.uk/repository/handle/10871/137456.

## Supplementary Materials

The following supporting information can be downloaded at: https://www.mdpi.com/article/10.3390/antiox13091124/s1, Figure S1: The relationship between MDA concentration in the ejaculate of fathers and six month survival of offspring.
