# Supplementary material for: Variation in Lipid Peroxidation in the Ejaculates of Wild Banded Mongooses (Mungos mungo): A Test of the Oxidative Shielding Hypothesis
Source: Antioxidants (Basel). 2024 Sep 18;13(9):1124. doi: 10.3390/antiox13091124 (PMC11429081; doi:10.3390/antiox13091124)
Supplement: Supplementary file 1 [file antioxidants-13-01124-s001.zip › antioxidants-3213568-supplementary.pdf]

Supplementary intergenerational data

Only 4 fathers for which we had MDA measures from their ejaculates were confirmed to sire offspring in the population, and only 3 of these fathers had MDA measures from both sampling periods. Out of 11 pups with confirmed paternities from these sampled males, 2 pups died before reaching 6 months of age; both were sired by the same male, HM293 (Figure S1). HM293 had the highest mean MDA concentration in their ejaculate of any of these 4 males, and the lowest reduction in MDA damage from the non-mating into the mating period. Of course, no formal analysis of these data is possible. We note that HM293 belonged to social group 1H while the other three males were from group 1B. 1H was a much smaller group that disintegrated a few years after sampling commenced. It is therefore possible that low quality of mothers and/or a poor rearing environment could have contributed to offspring mortality.

Additionally, ejaculate MDA measures were rarely taken during the proceeding mate competition event that led to the birth of these 11 offspring (3/11). For the purposes of presenting Figure S1 MDA levels were averaged across all sampling periods. This could mask any short-term variation in each father's oxidative status which could be important for survival of offspring. Overall, given these data deficiencies, it is not possible to determine whether there is an association between ejaculate MDA levels and offspring survival in the present study.

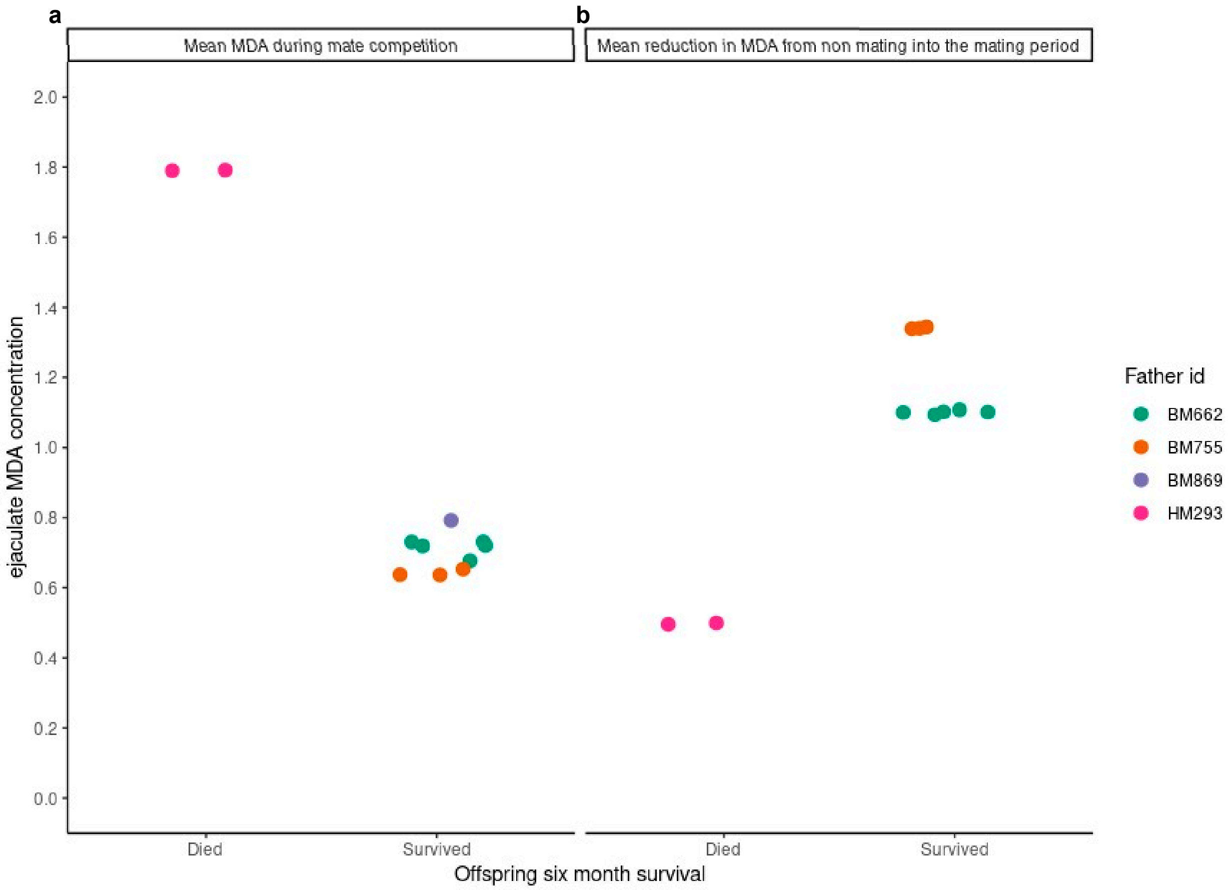

Figure S1: The relationship between MDA concentration in the ejaculate of fathers and six month survival of offspring. *MDA concentrations are given as a) the mean for all mate competition periods sampled for each father and b), as a measure of the strength of a shielding response, the mean reduction in MDA from the mean of all non-mating periods sampled to the mean of all mate competition periods sampled. Each point represents a pup that emerged from the den and had their father's id confirmed with pedigree data.*
